# Editorial: Recent advances in genetic and proteomic biomarkers involved in the early detection of solid tumors

**DOI:** 10.3389/fgene.2023.1260121

**Published:** 2023-09-06

**Authors:** Jitian Li, Apeng Chen, Ruowen Zhang, Changwei Li, Jian-Guo Zhou, Bin Fang, Yinan Jiang

**Affiliations:** ^1^ Henan Luoyang Orthopedic Hospital (Henan Prcovincial Orthopedic Hospital), Henan University of Chinese Medicine, Zhengzhou, China; ^2^ Lanzhou Veterinary Research Institute, Chinese Academy of Agricultural Sciences, Lanzhou, China; ^3^ Jiahehongsheng (Shenzhen) Health Industry Group, Shenzhen, China; ^4^ School of Medicine, Shanghai Jiao Tong University, Shanghai, China; ^5^ Department of Radiation Oncology, University of Erlangen Nuremberg, Erlangen, Germany; ^6^ The First Affiliated Hospital of Guangzhou University of Chinese Medicine, Guangzhou, China; ^7^ Children’s Hospital of Pittsburgh, School of Medicine, University of Pittsburgh Pittsburgh, Pittsburgh, United States

**Keywords:** solid tumor, biomarker, detection, treatment, prognosis

Cancer, as one of the leading causes of death, remains a huge challenge for public health worldwide. In clinics, early diagnosis and effective treatment can affect the prognosis of the patients. Moreover, favorable biomarkers and technology can assist in the early diagnosis, treatment, and prognosis of cancers. In this Research Topic, we accepted 8 articles, including 4 original research articles, 2 reviews, 1 case report, and 1 meta-analysis which covered the content of the Research Topic (*Recent advances in genetic and proteomic biomarkers involved in the early detection of solid tumors*).

Jiang et al. review the current advances in the identification, characterization, and validation of biomarkers of liquid biopsy for early solid tumors and roundly summarize the advantages and disadvantages of liquid biopsy. Also, they identify some limitations such as a single type and a small sample size which affect their diagnostic accuracy, sensitivity, and specificity. With the development of new assay technology, the detection methods need to be improved for identifying benign biomarkers in the early diagnosis of cancers. Reducing manual steps and increasing automation are the ultimate goals in tumor detection.

Consequently, finding new tumor indicators for diagnosis, prognosis, and treatment is crucial for cancer patients in the clinic. Generally, the tumor genome is hypomethylated. However, genome-wide DNA hypomethylation might lead to increased chromosomal instability and promote tumorigenesis. Li et al. show that alterant levels of Cathepsin Z (CTSZ) methylation in peripheral blood might be associated with breast cancer (BC), and CTSZ methylation might serve as a benign biomarker for identifying early-stage BC. As reported, lncRNA could control cancer-related gene expression through epigenetic, transcription, and post-transcription modification. Zhang et al. conduct a meta-analysis and bioinformatics analysis to explore the role of LINC00662 in human cancers. The result reveals that LINC00662 has irregular expression in various cancers. In addition, LINC00662 overexpression predicts earlier lymph node and distant metastasis. In conclusion, LINC00662 could be an early diagnostic marker to assist patients with early diagnosis and treatment. Initially, the cancerous inhibitor of protein phosphatase 2A (CIP2A) was reported as a tumor-associated antigen (known as p90). In this Research Topic, Chen et al. review the recent advances relating to CIP2A/p90 and their implications for future research. Notably, the autoantibody to CIP2A/p90 has a favorable diagnostic performance in the early detection of cancers.

With the development of various treatments, more and more effective drugs have been developed to assist early treatment of patients. Chonglou, a natural medicine, has been reported as an effective drug in inhibiting the development of various cancers. Moreover, Zhao et al. find that the active ingredients of Chonglou could inhibit the progression of lung adenocarcinoma (LUAD) cells. Furthermore, epithelial-mesenchymal transition (EMT) is closely associated with the progression of LUAD. Finally, 12 EMT-LUAD-Chonglou-related genes with five core drug targets are identified which effectively participated in the Chonglou treatment of LUAD.

Identified prognostic genes can regulate tumor growth and progression and assist early detection of cancers. Ferroptosis caused by iron-dependent lipid peroxides might play an important role in cancer. Huang et al. construct and explore the prognostic impact of a novel FRG-based osteosarcoma (OS) prognostic model. In the study, the model could distinguish the high- or low-risk group effectively which could provide a basis for the early detection of OS. Currently, metastatic complication is the leading cause of BC-related death. Based on this reason, it is of great significance to discover distant metastasis (DM)-related biomarkers or signatures related to DM-free survival (DMFS). Finally, Ren et al. identify seven genes (TSPAN7, CD74, MMP11, MELK, COL11A1, CHRDL1, and PITX1) and a signature (CD74 and TSPAN7) which might predict DMFS. Accurate prediction of DM would provide help for patients to receive early diagnosis and timely treatment.

Gene expression profiling could be used to trace the location of primary tumors and has been reported in various studies. The case report from Hu et al. highlights that identifying primary tumor location plays an important role in the treatment of unknown primary (CUP) cancer. The confirmation of the primary tumor location led to the early detection of CUP. Based on this, effective and targeted treatment plans are proposed.

In summary, the articles in this Research Topic summarize the recent advances in categories, techniques, and mechanisms of solid tumor biomarkers, especially genetic and proteomic biomarkers. Moreover, we thank all the authors and reviewers who contributed to the Research Topic. In the end, we hope the content of this Research Topic can provide useful advice for the study of solid tumors.

